# No Effect of 2 mA Anodal tDCS Over the M1 on Performance and Practice Effect on Grooved Pegboard Test and Trail Making Test B^1^,^2^,^3^

**DOI:** 10.1523/ENEURO.0072-14.2015

**Published:** 2015-08-31

**Authors:** Asbjørn J. Fagerlund, Janita L. Freili, Therese L. Danielsen, Per M. Aslaksen

**Affiliations:** Department of Psychology, Faculty of Health Sciences, The Arctic University of Norway, 9037 Tromsø, Norway

**Keywords:** cognition, motor speed, transcranial direct current stimulation

## Abstract

Previous studies suggest that transcranial direct current stimulation (tDCS) can facilitate motor performance and learning. In this double-blind experiment, 60 healthy human subjects (29 females) were randomized into three groups (active tDCS, sham tDCS, and no-treatment control group) in order to investigate the effect of a 20 min session of 2 mA tDCS over the motor cortex contralateral to the dominant hand on practice effect and performance on the Grooved Pegboard Test (GPT) and Trail Making Test (TMT). Performance was operationalized as the time to complete the tests before, during, and after stimulation. The practice effect was termed as the difference in time to complete the tests from pretest to post-test. Data on body mass index (BMI), head circumference, sleep status, interelectrode impedance, and caffeine and nicotine use were sampled to control for the influence of individual differences on the effect of tDCS. Adverse effects were registered using a standardized form. The results indicated no effect of tDCS on performance and practice effects on the GPT and TMT. For all groups, BMI was a predictor for a practice effect on the TMT. In the active tDCS group, high caffeine intake and low impedance predicted a practice effect on the GPT for the dominant hand. The present results suggest that impedance levels in tDCS studies should be routinely reported in future studies, as it might not only provide valuable information on the efficacy of the blinding conditions and participant discomfort, but also correlate with individual differences that are relevant to the outcome of the stimulation.

## Significance Statement

Transcranial direct current stimulation (tDCS) is a noninvasive brain stimulation technique that can modulate neuronal activation thresholds. The capability to enhance or diminish cortical excitability has previously been consistently demonstrated in experiments, with no serious adverse effects. The effect of tDCS on behavioral outcomes, especially on healthy subjects, appears less consistent. We tested whether stimulation over the motor cortex affected performance and practice effect on two commonly used neuropsychological tests that require fine motor skills, finger dexterity, and psychomotor speed. The results indicated no effect of the stimulation on these outcomes. Uniquely for subjects who received active tDCS, caffeine intake and electrode impedance predicted increased practice effect. The authors suggest that impedance levels should be routinely reported in tDCS studies.

## Introduction

Plasticity in the motor cortex (M1) plays an important role in motor skill learning (Ungerleider, 1995), and it is likely that a neurophysiological correlate of motor skills resides in the M1 (Nudo et al., 1996). As the acquisition of new motor skills are accompanied by changes in neuronal activity and excitability (Nitsche et al., 2003), it is therefore possible that transcranial direct current stimulation (tDCS) of the M1 can induce measurable changes in motor learning and performance. Results from several studies do indeed indicate that stimulation can enhance performance on motor tasks in healthy subjects. For instance, anodal tDCS over the left motor area improves right hand performance on a finger-sequencing task more than cathodal stimulation, but with opposite polarity effects for the left hand (Vines et al., 2006). Anodal tDCS over the M1 can also facilitate implicit learning on a serial reaction time task (Nitsche et al., 2003). In addition to polarity-specific effects, the timing of stimulation seems to be important, as anodal, but not cathodal, tDCS during an explicit learning task increases the rate of learning, but if applied prior to the learning task, both cathodal and anodal tDCS decrease the rate of learning (Stagg et al., 2011). In aged healthy subjects, anodal tDCS over the left M1 significantly improves right hand performance, compared to sham tDCS, on a task that mimics activities of daily living, including fine motor skills (Hummel et al., 2010). Furthermore, in older subjects, recent evidence indicates that anodal tDCS increases the retention of practice effect, compared to sham tDCS, on the grooved pegboard test (GPT) 35 min after the stimulation, but with no difference immediately following the stimulation (Parikh and Cole, 2014). In stroke patients, tDCS improves performance both on simple (Hummel et al., 2005) and more complex motor tasks (Fregni et al., 2005; Boggio et al., 2007). The variation in methodology makes the above results difficult to compare. However, a meta-analysis on polarity-specific effects of tDCS on behavioral motor and cognitive outcomes (Jacobson et al., 2012) indicate that the facilitation effect of anodal tDCS is more consistent than the inhibition effect of cathodal tDCS in studies with cognitive outcomes. Furthermore, results from another study (Cuypers et al., 2013) suggest that the effect sizes of anodal tDCS facilitation effects on learning tasks can be increased by increasing the stimulation intensity. Finally, there is also a high intersubject variability in the response to tDCS (Wiethoff et al., 2014). Participant age, interindividual differences in anatomy (Datta et al., 2012), and functional state of the brain, such as fatigue and activity (Silvanto et al., 2008; Antal et al., 2014), might affect the outcome of the stimulation. These factors are often not considered in studies that investigate the effect of tDCS on motor outcomes.

The aim of the present study is to test the effect of 2 mA anodal tDCS in healthy subjects. The present study tests performance and practice effects on two commonly used neuropsychological tests, which measure fine motor skills (GPT) and psychomotor speed [trail making test (TMT)] in an experimentally controlled study. In addition to a sham group, the present study included a no-treatment control group to estimate possible placebo effects (Benedetti et al., 2003), as the true placebo effect is represented by the difference between the sham and no treatment conditions (Fields and Levine, 1984).

It was hypothesized that the time to complete the tests would be lower during stimulation in the group that received active tDCS, compared to the sham tDCS and control groups. Furthermore, it was hypothesized that the practice effect, operationalized as the reduction in time to complete tests from pretest to post-test would be largest in the active tDCS group. Additionally, in order to investigate the influence of overt interindividual differences, body mass index (BMI) and head circumference were measured. The investigation correlates the functional state of the brain, sleep status, caffeine use, and nicotine use. Finally, electrode impedance was investigated as a possible predictor for practice effects in the active tDCS group.

## Materials and Methods

### Participants

Sixty healthy human subjects (29 females; demographic properties are displayed in Table 1) participated in the study. All participants were informed that the study investigated the effect of tDCS on fine motor ability. The exclusion criteria included severe psychiatric conditions, defined as bipolar disorder, severe depression, and schizophrenia. Additional exclusion criteria consisted of neurological conditions, developmental disorders, pregnancy, and drug abuse. No participants reported that they were receiving treatment with a medication that acted on the CNS. No subjects who declared interest in participating met the exclusion criteria. The study was approved by the Regional Committee for Medical and Health Research Ethics (2010/2256), and all participants gave their written informed consent in accordance with the Declaration of Helsinki guidelines.

**Table 1:** Mean values for demographic, head circumference, and behavioral measures

	Sham group	Active group	Control group	p*
Total *n* (female *n*)	20 (10)	20 (11)	20 (8)	0.64
Right handed (*n*)	19	19	18	0.77
Age (years)	23.65 (3.12)	24.10 (4.24)	23.80 (3.33)	0.92
BMI (kg/m^2^)	25.01 (4.10)	24.32 (2.89)	25.80 (4.55)	0.49
Head circumference (cm)	57.30 (2.01)	57.30 (1.47)	57.48 (1.61)	0.94
Sleep time (h)	6.95 (1.06)	6.95 (1.38)	6.78 (1.63)	0.90
Doses of nicotine (*n*)	0.65 (1.27)	0.60 (.88)	1.5 (2.01)	0.10
Cups of coffee (*n*)	0.60 (.82)	0.75 (1.29)	0.85 (1.01)	0.77
Awake time (h)	5.25 (2.29)	5.57 (3.42)	4.10 (2.44)	0.22
Impedance (kΩ)	NA	5.01 (1.09)	NA	NA
No nicotine doses (*n*)	14	13	9	NA
No coffee (*n*)	11	12	9	NA

Values are reported as the mean (SD), unless otherwise indicated. Frequencies for participants who did not consume nicotine and caffeine 2 h before the experiment are displayed. NA, Not applicable.

*p Value for one-way ANOVA for variable × group interaction.

### Study design

The study was designed as a double-blind study (single-blind study for the control group) with the following three groups: active tDCS group, sham tDCS group, and natural history control group with three repeated measures (RM; T1 pretest; T2 during stimulation, T3 post-test).

### Randomization and blinding

The participants were randomized into the following three groups: active tDCS group, 20 participants; sham tDCS group, 20 participants; control group, 20 participants). Every third participant was randomized to the control group by order of inclusion. The DC stimulator was set up for study mode, and was started by entering a 5 digit code in the display. Allocation to the active or sham tDCS group was performed by assigning each participant to an individual 5 digit code from a list consisting of 40 codes, where 20 were associated with active tDCS and 20 were associated with sham tDCS. The order of the treatment codes was randomized using a random number generator (randomize.org). The key to the individual treatment codes was kept separate from the experimenters, and thus the active and sham conditions were double blind.

### tDCS

tDCS was administered using a neuroConn DC Stimulator, a battery-driven device that constantly monitors electrical impedance and terminates the stimulation if the voltage exceeds safety limits. The stimulation duration was 20 min with an intensity of 2 mA. DC was transferred by a pair of 35 cm^2^ (0.057 mA/cm^2^) rubber electrodes inserted into sponge pads soaked with 10 ml of medical grade sterile water. Electrode sponges were changed to a clean pair between participants. To reduce the skin sensation and achieve improved connection on the electrode–scalp interface, Ten20 neurodiagnostic electrode paste (Weaver and Company) was applied to the scalp at the site of stimulation. The electrode positioning was similar to that in the study by Fregni et al. (2006). In order to stimulate M1, the anode was placed at the C3 or C4 positions in the 10/20 system for EEG electrode positions, on the position contralateral to the dominant hand. The cathode was placed in the supraorbital area, contralateral to the anode. To reduce discomfort, the stimulation had fade-in and fade-out periods of 20 s. Sham tDCS consisted of an 8 s fade-in, followed by 30 s of DC stimulation, and was terminated by a 5 s fade-out. The sham condition mimicked the skin sensation of active tDCS, but had insufficient duration to induce aftereffects in cortical excitability (Nitsche and Paulus, 2000).

### Outcomes

#### Grooved pegboard test

The grooved pegboard test (Lafayette Instrument) was used to test fine motor speed, visuomotor speed, and eye–hand coordination. The test consists of a board with 25 keyholes that requires keys to be correctly rotated and inserted. The keys are located on a tray above the keyholes. The participants were instructed to complete the test as fast as possible, and the outcome was evaluated in seconds from the start to all 25 keys correctly placed. The GPT was administered for the dominant hand (GPD) and for the nondominant hand (GPN) at all three time points.

#### Trail making test

In order to test whether there were changes in dual and divided attention abilities, attention shift, and psychomotor speed, the TMT B (Halstead–Reitan Neuropsychological Battery) was used. The test consists of drawing a line between numbered points on a paper in the correct sequence. The participants were instructed to complete the test as fast as possible, and the outcome was evaluated in seconds from start to completion.

#### Individual differences

Before starting the study, the head circumference of the participants was measured. The BMI of the participants was calculated by measuring their weight and height. Hand dominance was registered by participant self-report. Sleep status was registered as the number of hours of sleep for the participant on the night before undergoing the test, and the number of hours awake at the time of testing. Caffeine and nicotine (cigarettes or chewing tobacco) use was registered as the number of doses in the 2 h preceding the study. Electric impedance (in kilo-ohm) was registered by reading the value in the stimulator display after 1 min of stimulation. In the sham condition, a random impedance value was displayed, and thus sham and active stimulation appeared similar to the experimenters.

#### Adverse effects

The adverse effects in the active and sham groups were registered using a structured interview (Brunoni et al., 2011) following each session. The participants were asked to report whether they had a headache, scalp pain, tingling, itching, burning sensation under electrodes, sleepiness, trouble concentrating, acute mood change, and other adverse effects after the stimulation. The redness of the skin was evaluated by the experimenter. The intensity of the adverse effects was coded as follows: 0, none; 1, mild; 3, moderate; and 4, intense.

### Procedure

**Figure 1.**
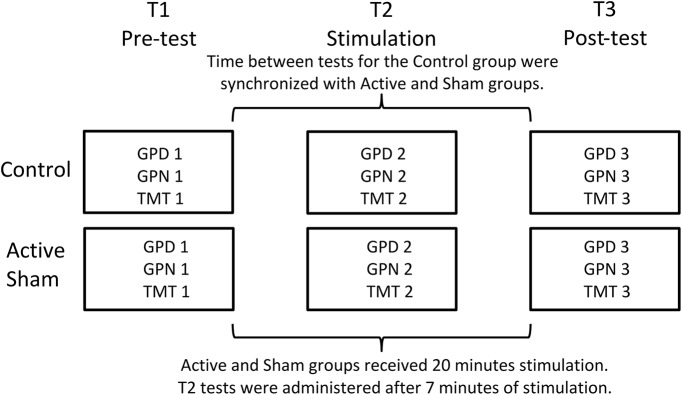
Overview of the experimental procedure. The control group followed the same procedure as the active and sham groups, but without the electrode montage. Stimulation started immediately after tests at T1 were completed. Tests at T2 were administered after 7 min of stimulation. Tests at T3 were administered immediately after the stimulation was completed.

**Figure 2.**
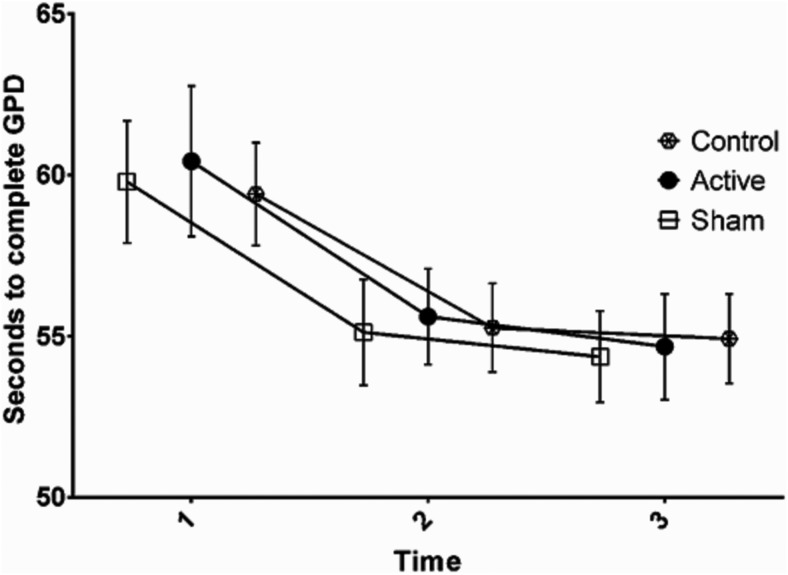
Seconds to complete GPD at pretest (1), during stimulation (2), and post-test (3). Error bars denote the SEM.

**Figure 3.**
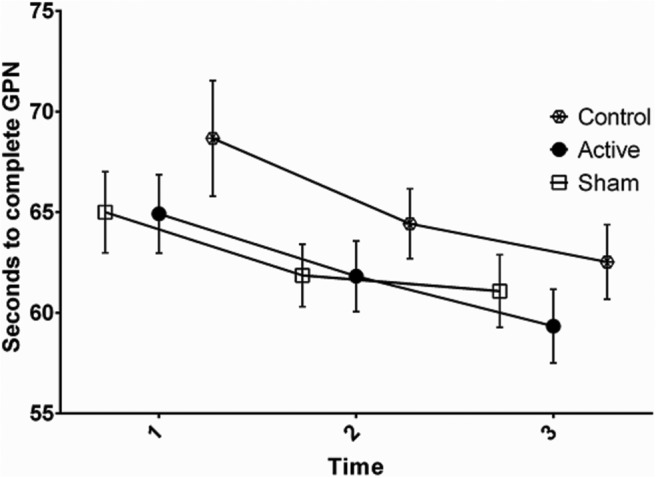
Seconds to complete GPN at pretest (1), during stimulation (2), and post-test (3). Error bars denote the SEM.

Prior to conducting the study, the participants were screened for exclusion criteria, and the control data were obtained (Fig. 1). All trials in the study were conducted by the same two female experimenters in a soundproof laboratory with thermostatic controlled temperature. The control group underwent the same procedure as the active and sham groups, without the electrode montage. For the active and sham groups, the electrodes were mounted on the scalp, and the participants performed the pretest GPD (GPD1), GPN (GPN1), and TMT (TMT1; Figs. 2, 3). After completing the pretest, the tDCS was started, and after 1 min the impedance (in kilo-ohm), as indicated in the stimulator display, was registered. After 7 min of stimulation, the participants performed the tests under stimulation (GPD2, GPN2, TMT2). Finally, after the 20 min stimulation was complete, the participants performed the post-test (GPD3, GPN3, and TMT3). For the control group, the timing of the test administration was synchronized with that of the active and sham groups so that the total duration of the study was similar across all three groups. Adverse effects from the stimulation were registered in the active and sham groups immediately after the study using a structured interview.

### Statistical analysis

SPSS version 22 (IBM) was used for inferential analyses, and *post hoc* power calculations that were performed in G*Power version 3.1.9.2 (Heinrich-Heine-Universität, Düsseldorf, Germany). To test the interactions among three levels of group (active, sham, and control) and three levels of time (T1, pretest; T2, stimulation; T3, post-test), an RM ANOVA was used. Paired-samples Bonferroni-corrected *post hoc t* tests were applied to investigate between-group differences when appropriate. To investigate the influence of control variables of the practice effect on GPT and TMT, we conducted partial correlation analyses, and further investigated variables that significantly correlated with practice effect using multiple linear backward regression with the control variables as predictors and change score (pretest − post-test) as the dependent variable. One-sample Kolmogorov–Smirnov tests were used to test the normal distribution of the variables. For the RM ANOVA, the effect sizes were calculated as partial η^2^ (η^2^*_p_*). For the multiple linear regression effect, sizes were calculated as *R* ^2^. Mauchly’s test was used to test the assumption of sphericity between the conditions. Alpha levels were set to ≤0.05.

## Results

Prior to analyzing the effect of tDCS on the outcome measures, we compared the pretest results among the three groups using one-way ANOVA to eliminate the possibility that the differences observed after tDCS were due to the groups being different at baseline. One-way ANOVA indicated that the three groups had similar performances on the GPD (*F*_(2,59)_ = 0.07, *p =* 0.93^a^), GPN (*F*_(2,59)_ = 0.93, p = 0.40^b^ ) and TMT (*F*_(2,59)_ = 0.21, *p =* 0.81^c^) in the pretest. The data on GPD, GPN, and TMT were normally distributed (see Table 5, for definition of superscript designations).

### Effects of tDCS on GPT

On the GPD test, there was a significant effect of time (*F*_(2,114)_ = 39.76, *p* < 0.01, η^2^*_p_* = 0.41^d^), but no significant group × time interaction term was observed (*F*_(4,114)_ = 0.18, *p* = 0.95, η^2^*_p_* = 0.01^e^). On the GPN test, there was a significant effect of time (*F*_(2,114)_ = 20.04, *p* < 0.01, η^2^*_p_* = 0.26^f^), but no significant group × time interaction term was observed (*F*_(4,114)_ = 0.42, *p* = 0.79, η^2^*_p_* = 0.41^g^). The results indicated that while there was a general reduction in the number of seconds to needed to complete the GPD and GPN from pretest to post-test, there were no between-group differences.

### Effects of tDCS on TMT

**Figure 4.**
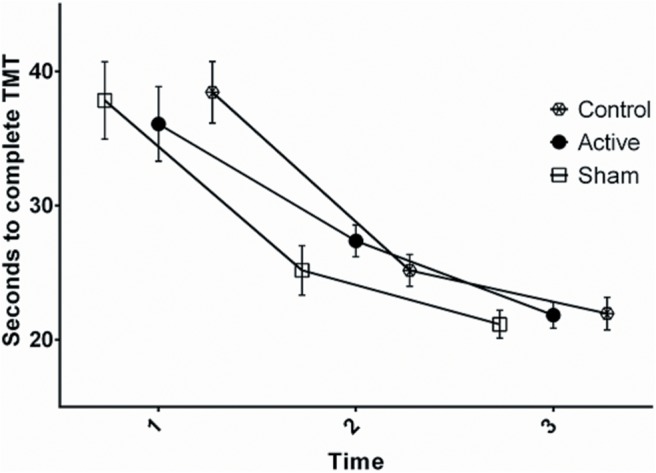
Seconds to complete TMT at pretest (1), during stimulation (2), and post-test (3). Error bars denote the SEM.

For the TMT (Fig. 4), the degrees of freedom were corrected using Greenhouse–Geisser estimates of sphericity (*ε* = 0.71). The results show that the number of seconds needed to complete the test was reduced over time (*F*_(2,114)_ = 101.55, *p* < 0.01, η^2^*_p_* = 0.64^h^), but no significant group × time interaction term (*F*_(4,114)_ = 0.79, *p* = 0.50, η^2^*_p_* = 0.03^i^) was observed.

### Individual differences

On the Δ scores (T1 − T3), one-way ANOVA indicated there were no significant differences among the groups on the GPD (*F*_(2,57)_ = 0.27, *p* = 0.76^j^), GPN (*F*_(2,57)_ = 0.52, *p =* 0.59^k^), and TMT (*F*_(2,57)_ = 0.26, *p* = 0.77^l^). Furthermore, there were no differences between sham and control group T1 − T3 scores on the GPD (*F*_(1,39)_ = 0.44, *p =* 0.51^m^), GPN (*F*_(1,39)_ = 0.82, *p* = 0.37^n^), and TMT (*F*_(1,39)_ = 0.01, *p* = 0.94^°^), indicating no significant placebo effect. Because of the large observed unsystematic variance caused by naturally occurring individual differences in practice effect, compared to the variances that were a systematic effect of the experimental manipulation in the ANOVA, we conducted an exploratory partial correlation analysis with gender as a control variable to investigate the influence of individual differences on the T1 − T3 scores for the GPD, GPN, and TMT^p^. There was a positive correlation between BMI and TMT T1 − T3 (*r* = 0.28, two-tailed *p =* 0.03). Since the impedance variable only was viable for the active tDCS group, a similar analysis was conducted, filtering out the Sham and Control groups, and included impedance^q^. In the active group, there were correlations between GPD T1 − T3 and the number of caffeine doses the last 2 h (*r* = −0.56, two-tailed *p* = 0.01), and impedance (*r =* −0.64, two-tailed *p* < 0.01). For GPN T1 − T3, no significant correlations with the individual difference variables were identified.

To further investigate the influence of variance in the significantly correlated (*p* < 0.05) control variables on the T1 − T3 scores, we conducted two linear regression analyses: regression 1 with TMT T1 − T3 as the dependent variable, and BMI, gender, and dummy-coded group affiliation for the sham and control groups as predictors^r^; and regression 2 for the active group with GPD T1 − T3 as the dependent variable, and gender, number of caffeine doses the last 2 h before the test, and impedance as predictors^s^ (Tables 2, 3).

**Table 2:** Mean values for GPT with dominant and non-dominant hand (GPD / GPN) and TMT with confidence intervals on 1 pre-test, 2 during stimulation and 3 post-test, and T1-T3 Δ scores with 95% confidence intervals

	Sham group		Active group		Control group	
Mean	Mean	95% CI	Mean	95% CI	Mean	95% CI
GPD1	59.79	55.82–63.76	60.43	55.55–65.32	59.41	56.07–62.74
GPD2	55.12	51.69–58.55	55.61	52.20–58.72	55.26	52.38–58.14
GPD3	54.36	51.39–57.34	54.68	51.25–58.11	54.92	52.00–57.84
GPN1	65.00	60.78–69.21	64.62	60.54–68.70	68.67	62.67–74.68
GPN2	61.86	58.60–65.11	61.82	58.15–65.49	64.43	60.79–68.08
GPN3	61.08	57.30–64.85	59.34	55.51–63.18	62.53	58.65–66.41
TMT1	37.84	31.84–43.87	36.09	30.30–41.88	38.45	33.66–43.24
TMT2	25.18	21.33–29.04	27.37	24.89–29.85	25.17	22.69–27.65
TMT3	21.60	19.41–23.79	21.84	19.79–23.88	21.96	19.42–24.51
GPD Δ	5.42	3.11–7.73	5.76	2.31–9.20	4.49	2.63–6.35
GPN Δ	3.92	0.66–7.18	5.28	2.95–7.61	6.14	2.18–10.11
TMT Δ	16.24	11.34–21.14	14.26	8.90–19.61	16.49	11.65–21.33

**Table 3:** Statistics for regressions 1 and 2

	*B*	*t*	*β*	*p*	Partial correlation
Regression 1					
Included variables					
BMI	0.77	2.26	0.29	0.03*	
Excluded variables					
Sham	0.04	0.30		0.76	0.04
Control	0.02	0.14		0.89	0.02
Gender	−0.15	−1.23		0.22	−0.16
Regression 2					
Included variables					
Gender	3.28	2.01	0.27	0.52	
Impedance	−1.77	−2.46	−0.32	0.02*	
Caffeine intake	2.11	0.75	0.37	0.01**	

The results from regression 1 indicate that BMI, but not group affiliation and gender, was a predictor for an increased practice effect on the TMT. The results from regression 2 indicate that, for participants who had received active tDCS, caffeine intake prior to participating in the study and electrode impedance were predictors for increased practice effects on the GPD. There was also a tendency toward male participants having an increased practice effect, but this did not reach significance.

### Adverse effects

In general, the stimulation was well tolerated by the participants, and no sessions were aborted due to adverse effects. The occurrence of adverse effects in the active and sham groups, and the mean difference in intensity between groups are displayed in table 4 (Table 4). The sensation of burning under the electrodes was significantly more intense in the active group, compared with the sham group, with a similar tendency for skin redness. In the active group, an exploratory correlation analysis was conducted to investigate the relation between impedance and the registered adverse effects. No correlations were significant (*p* < 0.05).

**Table 4:** Frequency of sessions after which specific adverse effect occurred, and the mean intensity for specific adverse effects across all sessions defined as 0 = none, 1 = mild, 2 = moderate, 3 = severe.

	Frequency by sessions		Mean intensity (SD)		
Sham group (*n* =20)	Active group (*n* = 20)	Sham group	Active group	Sham group	p*
Headache	6	2	0.60 (.94)	0.20 (.62)	0.120
Neck pain	2	0	0.20 (.62)	0	0.154
Scalp pain	3	3	0.40 (.99)	0.40 (1.05)	1
Tingling	8	8	0.80 (1.01)	0.80 (1.01)	1
Itching	5	6	0.55 (1.00)	0.75 (1.25)	0.580
Burning sensation	6	13	0.7 (1.13)	1.45 (1.19)	0.048**
Skin redness	11	17	1.25 (1.25)	1.95 (.94)	0.053
Sleepiness	9	6	1.00 (1.21)	0.85 (1.42)	0.722
Trouble concentrating	1	3	0.10 (.45)	0.40 (.99)	0.226
Acute mood change	0	0	0	0	1
Others	1	1	.10 (.45)	.30 (.73)	0.304

*p-value for independent samples t-test for group differences in adverse effect intensity.

**p < .05.

**Table 5:** Statistics

		Data structure	Type of test	Power (%)
a	GPD	Normally distributed	One-way ANOVA	6
b	GPN	Normally distributed	One-way ANOVA	20
c	TMT	Normally distributed	One-way ANOVA	8
d	GPD RM time	Normally distributed	RM ANOVA	100
e	GPD*group	Normally distributed	RM ANOVA	9
f	GPN RM time	Normally distributed	RM ANOVA	100
g	GPN*group	Normally distributed	RM ANOVA	13
h	TMT RM time	Normally distributed	RM ANOVA	100
i	TMT*group	Normally distributed	RM ANOVA	9
j	Δ GPD	Normally distributed	One-way ANOVA	9
k	Δ GPN	Normally distributed	One-way ANOVA	13
l	Δ TMT	Normally distributed	One-way ANOVA	15
m	Δ GPD (sham and control)	Normally distributed	One-way ANOVA	81
n	Δ GPN (sham and control)	Normally distributed	One-way ANOVA	99
o	Δ TMT (sham and control)	Normally distributed	One-way ANOVA	12
p	Corr all	Non-normally distributed	Partial Pearson correlation, two-tailed	65
q	Corr active	Non-normally distributed	Partial Pearson correlation, two-tailed	25
r	Regression all	Non-normally distributed*	Linear multiple regression	97
s	Regression active	Non-normally distributed*	Linear multiple regression	41

Lines refer to the alphabetical value provided in the Results section. *Post hoc* power calculations were performed on the sampled data in G*Power for Windows. For one-way and RM ANOVA, the *n* and SDs from the data with desired alpha level of 0.05 were used. For the correlation analysis, a hypothetical regression value of 0.03 against a zero correlation were used. For regression analysis, the observed *R^2^* values were used. Deviations from normal distribution in the sample containing all participants were significantly non-normal: head circumference, *D*(60) = 0.15, *p <* 0.01; age, *D*(60) = 0.22, *p* < 0.01; doses of nicotine in last 2 h before undergoing stimulation, *D*(60) = 0.33, *p <* 0.01; doses of caffeine in last 2 h before undergoing stimulation, *D*(60) = 0.29, *p* < 0.01; hours awake, *D*(60) = . , *p* = 0.02; and hours of sleep, *D*(60) = 0.15, *p* < 0.01. Deviations from normal distribution in the sample containing participants from the active group were significantly non-normal: age, *D*(20) = 0.21, *p* = 0.02; doses of nicotine in last 2 h before undergoing stimulation, *D*(20) = 0.40, *p* < 0.01; doses of caffeine, *D*(20) = 0.32, *p <* 0.10; and hours of sleep, *D*(20) = 0.21, *p* = 0.02.

*The assumptions for regression regarding homoscedasticity, independent errors and normally distributed errors were met.

## Discussion

The present study tested the effect of anodal 2 mA tDCS over the M1 on performance and practice effects in two commonly used neuropsychological tests, while controlling for the influence of interindividual differences in anatomy and functional state of the brain.

### Effect of tDCS on performance and practice effect on GPT and TMT

The results indicated no effect of a 20 min session of anodal 2 mA tDCS over the M1 on performance and practice effects on the GPD and GPN, and on the TMT, compared with the sham and control groups. The active, sham, and control groups had equal performance before, during, and after stimulation. Furthermore, no placebo effect represented as the difference between the sham and the control groups was observed.

A consensus article stated that there are no aftereffects of tDCS found on GPT results (Ziemann et al., 2008). In the present study, it was hypothesized that the increased intensity of stimulation would produce stimulation effects that surpassed those of earlier studies. However, there were no effects of 2 mA 20 min anodal tDCS on performance or practice effect. An increased retention of a practice effect, which recently was demonstrated on older subjects after 1 mA stimulation (Parikh and Cole, 2014), cannot be ruled out, as the retention of a practice effect was not measured in the present study. The TMT is a commonly used neuropsychologic test that is sensitive for organic brain injury (Reitan, 1958) and can also serve as a measure for general psychomotor speed in healthy individuals. In the present study, the anodal stimulation of M1 had no effect on the performance and practice effect of this test. The results are similar to the findings in a recent study (Park et al., 2014) that applied bilateral frontal anodal tDCS (two stimulators) with extraencephalic cathodes on older subjects, and that also found no improvement on the TMT. However, in this study, the stimulation paired with computer-assisted training did improve verbal working memory. Consequently, it is possible that the TMT practice effect cannot be effectively improved by anodal tDCS, neither frontally nor over the motor cortex. In chronic stroke patients, anodal tDCS over the ipsilesional M1 has been shown to improve performance on specific motor tasks (Fregni et al., 2005; Hummel et al., 2005; Boggio et al., 2007). However, a more recent study (Rossi et al., 2013) on acute stroke patients failed to demonstrate effects on clinically relevant recovery. It is likely that the excitatory effects of anodal tDCS may produce different behavioral outcomes in healthy and impaired subjects. The participants in the present study were university students and had high test performances at pretest on the GPT (Table 2) compared with the normative group with ≥13 years of education (Ruff and Parker, 1993). If a ceiling effect on the test were present, it may have left limited room for improvement, and thus reduced the observed practice effect.

### Effect of control variables on practice effects in GPT and TMT

For the entire sample, the results indicated that BMI was a weak predictor for practice effect on TMT where subjects with higher BMI achieving a slightly larger reduction in time to complete the test from pretest to post-test. As gender was controlled for in the analysis, further attempts to explain this novel finding would be difficult without going outside the scope of this report. More interesting with regard to the hypothesis, when analyzing only participants who had undergone active tDCS, there were two significant predictors for increased practice effect in GPD, as follows: lower impedance values and higher caffeine intake prior to the study. However, these effects were not present in the GPN and TMT, and thus cannot be regarded as general effects. There was a tendency toward males achieving a larger practice effect, but this did not reach significance.

In the present study, lower electrode impedance predicted an increased practice effect on the GPD. The predictive value of impedance on individual treatment outcomes have, to our knowledge, not been reported previously, and impedance levels are rarely reported or discussed in the literature. In tDCS, the target current intensity (in milliamperes) is set according to the stimulation protocol, and is thus a static value. The interelectrode impedance (in kilo-ohms) is the resistance in the flow path of electrons between the anode and the cathode, and is subject to individual differences. In the present study, a stimulator with automatic current control was used that automatically adjusted the voltage as per Ohm’s law. Thus, participants with higher interelectrode impedance required increased voltage in order to drive the current. It has been demonstrated that high impedance values, and consequently increased voltage, lead to an increased risk of skin lesions after consecutive sessions of tDCS (Palm et al., 2008; Frank et al., 2010). The voltage has been shown to be a determinant for discomfort under the electrodes during stimulation, with a threshold of ∼10 V (Lang et al., 2005). In addition to discomfort, skin sensation under the electrodes can reduce the efficacy of patient blinding. This has been reported to be the case at stimulation intensities of 2 mA (O'Connell et al., 2012). As per Ohm’s law, the voltage required to drive the current though a given impedance increases with increased intensity, and thus the risk of exceeding the threshold of 10 V. However, interelectrode impedance in human subjects is both time and current dependent, with peak values during the fade-in phase, with a decrease after the fade-in phase and incremental reduction throughout the stimulation period (Hahn et al., 2013). Furthermore, the factors that influence skin sensation under the electrodes are complex and not linearly or solely determined by voltage (Dundas et al., 2007). The present study used a current of 2 mA, and observed a mean impedance of 5.01 kΩ, resulting in a mean voltage of 10.02 V. The fade-in phase was 20 s, and the impedance levels were registered after 60 s, a time at which impedance levels were relatively stable. An exploratory correlation analysis indicated no significant correlation between impedance and the intensity of registered adverse effects, including tingling, scalp pain, and burning sensation under electrodes, indicating that the voltage did not exceed the threshold for discomfort. However, the registration form that was used in this study was designed for clinical adverse effects and might not be sensitive for discomforts at the lower end of the spectrum.

The results from the present study indicated that lower impedance predicted higher practice effect on the GPD. These results are difficult to interpret, as, to our knowledge, the relationship between electrode impedance and behavioral outcomes has not previously been demonstrated. Sulcus–gyro morphology in the human brain is complex and subject to large interindividual variations (Mangin et al., 2004), and is likely to affect the distribution of electric current in the brain during tDCS (Datta et al., 2012) and possibly also the electric resistance between the electrodes in tDCS. Thus, the observed individual differences in impedance in the present study may have correlated with individual differences in skull and brain anatomy that were relevant to the effect of stimulation on the practice effect in the GPD.

In the present study, neither tDCS nor caffeine intake predicted an increased practice effect in the GPD on the sample as a whole, but increased caffeine intake 2 h before the study predicted an increased practice effect in the active tDCS group, but not in the sham and control groups. Caffeine has an effect on a range on behavioral outcomes such as alertness, reduced fatigue, and performance on simple tasks (Smith, 2002), but not on the GPT specifically (Lieberman et al., 1987). It is possible that, in the present study, the cumulative effect of tDCS induced excitability increase, and caffeine induced increase in alertness, might have exceeded the threshold required to facilitate the practice effect. 

The effect of gender on excitability changes after tDCS have previously been described in the literature. Regarding changes in motor cortex neuroplasticity following tDCS, women had more immediate and prolonged inhibition following cathodal stimulation (Kuo et al., 2006), but there were no gender differences in excitability following anodal stimulation. Comparatively, regarding neuroplasticity in the visual cortex, no effect of cathodal stimulation was found, but a gender-specific effect of anodal stimulation occurred with immediate and prolonged facilitation in female participants, and a prolonged inhibitory effect in male participants (Chaieb et al., 2008). Considering these studies, the gender-specific effects of tDCS appear to be site specific. Regarding behavioral outcomes on motor performance and practice effect after tDCS, the results from the present study indicated a tendency toward a larger practice effect on the GPD for males compared to females in the active tDCS group. This tendency was not observed in the sham and control groups, and not on the GPN and TMT.

### Limitations

The stimulation in this study was delivered using two sponge electrodes, arranged in the relatively common M1–SO montage. Evidence from MRI-derived computer head models has indicated that the distribution of the electric field in the brain using this method is not focal (Mendonca et al., 2011); therefore, the hypothesis with which we applied anodal tDCS to the M1 might have been insufficiently precise with regard to the stimulation pattern. A high-definition tDCS technique would have made the distribution of electric field less widespread. However, the superiority of this method in terms of behavioral outcomes has not yet been demonstrated (Kuo et al., 2013). Therefore, it was considered interesting to test the effect of the relatively easy to apply M1–SO montage on neuropsychological tests with predictive value for real-life functional outcomes. The interpretation of the results may have been further complicated by the fact that the cathode was placed on the supraorbital area contralateral to the anode. It is therefore likely that the frontal cortex was under the influence of cathodal tDCS, which is known to produce inhibitory effects (Nitsche and Paulus, 2001). Furthermore, as the post-tests were performed immediately after the stimulation ended, the present study did not investigate the aftereffects or long-lasting effects of the stimulation. Future studies could investigate the prolonged effects of tDCS on motor performance as a recent study (Parikh and Cole, 2014) observed an increased retention of practice effect on the GPT after stimulation with a lower intensity than that used in the present study. Furthermore, the GPT and TMT were administered in the same order in every trial and in every group. Therefore, the order of the tests might have systematically affected the outcomes. Nicotine use in the present study was registered as “doses of nicotine” (any type of tobacco for oral intake), and thus the habitual consumption pattern, or whether the participants were under the influence of withdrawal effects, was not controlled for. Finally, the participant’s habitual pattern of caffeine consumption was not controlled for in the analyses. High and low habitual caffeine consumers may have responded differently to both intake and abstinence (Rogers et al., 2013), and thus the effect of caffeine intake 2 h before the experiment on the outcomes should be interpreted with caution.

### Conclusion

Contrary to the hypothesis, the stimulation had no effect on performance and practice effect on GPT and TMT. However, uniquely for the participants who received active tDCS, caffeine intake in the last 2 h before the study and lower electrode impedance predicted a larger practice effect for the GPD. Based on the current study, it is recommended that future studies that use tDCS on human subjects report electrode impedance, in addition to stimulation parameters and the method of electrode preparation. This may, as the present results suggests, affect the outcomes of the stimulation.

## Synthesis

The decision was a result of the Reviewing Editor Christophe Bernard and the peer reviewers coming together and discussing their recommendations until a consensus was reached. A fact-based synthesis statement explaining their decision and outlining what is needed to prepare a revision is listed below. The following reviewers agreed to reveal their identity: Michael Nitsche, Patrick Ragert

Both reviewers agree that your manuscript brings interesting results about tDCS. However, one important control experiment must be performed before considering further the manuscript: determining whether or not a cathode-dependent negative effect does not cancel an anode-dependent positive effect.

There are other major issues, which would require further analysis of the existing dataset. In addition, both reviewers indicate the need of discussing many issues in the manuscript.

Reviewer 1

Major

1. tDCS: Since both electrodes can presumed to be active in this electrode arrangement, and prefrontal areas are involved in both tasks, it might have been the case that the prefrontally situated cathode had an - possibly worsening - effect on performance, compensating for the effect of anodal tDCS. A control experiment excluding this, as suggested also by the authors in the discussion, might be helpful to rule out this possibility.

2. tDCS: The C3 electrode position is not contralateral to the dominant hand for all subjects, because some subjects were not right-handed (table 1). This statement should be corrected, and also mentioned as a minor limitation.

3. Discussion, general: There are some potential reasons why stimulation did have no effects on performance in these specific tasks, and I agree with the authors that tone probable explanation might be a ceiling effect. This argument might be supported also by the study of Park et al. 2014, who did not find effects in these, but other tasks in stroke patients undergoing tDCS. Another option might be that the primary motor cortex is not the ideal stimulation area for these tasks, which require also cognitive processes which are controlled by prefrontal areas. It would be helpful to refer in the introduction to areas involved in these tasks, and to discuss this topic appropriately.

4. Discussion, page 9: most of the studies cited to explain the results are studies in clinical populations. The authors might want to discuss a bit more other motor function tDCS studies, which were conducted in healthy humans.

5. Line 311ff: Could it be possible that impedance is correlated with BMI? If yes, this could hint for altered current flow caused by skull factors. A respective calculation might help to explain this effect.

6. Conclusion: since the authors state in the discussion that impedance was not associated with discomfort, the statement made in the conclusion about enhanced discomfort, and compromised blinding caused by high impedance seems not to be justified.

Minor

1. line 35f: "a motor skill" or "motor skills". Please correct! Other minor grammatical errors throughout the manuscript. Please proof-read carefully!

2. Line 56 ff: This sentence is difficult to understand. Do the authors mean motor cortex physiology by "motor outcomes"? This sentence should be re-phrased. Moreover, taking for granted that excitability/activity enhancement should always improve performance and vice versa, is an over-simplification, which was shown to be erroneous already in relatively early tDCS studies (e.g. Antal et al. J Cog Neurosci 2004). The effect of tDCS depends not only on its physiological impact, but also on task characteristics.

3. Line 60f: Please substitute the conference abstract of Leenus et al. by the respective published paper.

4. Line 84f, table 1: I presume that none of the participants did consume CNS-acting medication. Please add this statement, if it applies. Please add information how many subjects were smokers, because nicotine and nicotine withdrawal can relevantly affect the impact of tDCS on cortical excitability (Grundey et al. J Neurosci 2011). Smoking should also be reported in table 1.

5. Line 108ff: was 10 ml water adequate to soak the sponges, but not to moisten skin neighbored to the electrode, which would have resulted in a larger contact area, and thus reduce current density?

6. Line 123: 4 seconds are sufficient to induce excitability alterations (Nitsche &amp; Paulus 2000). I guess the authors want to refer to after-effects.

7. Line 266ff: An important difference between the studies conducted by Hummel et al. and Rossi et al. is that Hummel explored chronic, while Rossi acute stroke patients. This might well explain the difference of results between these studies (much more dynamic restitution independent from stimulation in acute stroke). I am not quite sure, how the results of the Rossi study can explain the results of the present study.

8. Line 307f: Please state the result of this correlation analysis in the results section too.

9. Table 5: This table is very difficult to read. It might be better to substitute the letters by content.

Reviewer 2

Major comments

1. As far as I can see, the order within/ between tests was not randomized. This might have affect the outcome of the study (order effect). This limitation should be discussed more thoroughly. Additionally, I wonder why GPD was tested with both hands, while TMT was only tested with one hand? Please explain.

2. Some crucial comparisons are missing in the manuscript. For example, the authors should perform an ANOVA to compare the sham and control group separately, which has not been done so far.

3. Interpretations of result: BMI seems to be a predictor for TMT performance. The reviewer wonders why this should be true for the other test (GPT)? The authors should discuss this issue in more detail. In a similar vein, why should caffein intake/ impedance only affect performance of the dominant hand in the GPT?! This seems surprising and needs further explanation. Due to the lack of general effects, I would suggest to tone down the statement that these factors might affect performance since they do not affect performance equally.

Minor:

1. check grammar/ typos throughout manuscript (e.g. page 2, line 38, line 62 etc.)

2. I suggest adding the following paper to the introduction which shows high inter-individual differences in response to tDCS:

"Variability in response to transcranial direct current stimulation of the motor cortex.

Wiethoff S, Hamada M, Rothwell JC.

Brain Stimul. 2014 May-Jun;7(3):468-75. doi: 10.1016/j.brs.2014.02.003. Epub 2014 Feb 15."

3. Nicotine use has been mentioned but the info is missing in table 1. Furthermore, caffeine use seems to be quite low. Please specify how many subjects did not intake caffein. This information is crucial for the interpretation of result.

4. I suggest to change "anatomical differences" to "differences in head circumference" otherwise readers might think the authors looked at brain anatomical differences (which I also thought of after reading the abstract)! Same for functional differences. I suggest spelling out what you looked at is more straight forward (caffein, sleep etc.)

5. Please change "Neurocon" (page 4) to "Neuroconn"

6. Why were different fade in and fade out times for anodal and sham tDCS used?! Please specify.

7. Adverse effect: The authors asked to report if participant show sleepiness. When should participant report that? Was the interview done on the next day? Please specify.

8. Many correlations were performed. Were correlations corrected for multiple comparisons? If yes, please indicate threshold levels when a test was considered as significant.

STATEMENT OF CORRECTIONS TO MANUSCRIPT NUMBER eN-NWR-0072-14 "NO EFFECT "NO EFFECT OF 2MA ANODAL TDCS OVER THE M1 ON PERFORMANCE AND PRACTICE EFFECT ON GROOVED PEGBOARD TEST AND TRAIL MAKING TEST B" SUBMITTED TO eNEURO.

Dear Dr. Bernard,

We thank all reviewers for insightful comments that have improved the manuscript substantially. The points of concern raised by the reviewers are discussed in the order in which they appear in the reviews.

Reviewer 1

Major

1. I agree that "anodal tDCS over the M1" is more a common tDCS taxonomy, indicating the most common electrode montage, and not a proper description of the distribution of electric field in the brain and the following effects on cerebral tissue excitability.

However, TMS verified alternations in Motor Evoked Potential (MEP) thresholds following anodal tDSC over the M1 [1, 2], that are subject to predictable pharmacological manipulation [3, 4], constitutes basic evidence for both the immediate excitatory effect of M1, as well as NMDA dependant after effects. Furthermore, the imaging evidence suggesting an important role of M1 in motor skill learning, as well as evidence for behavioral effects of anodal M1 tDCS that are presented in the introduction of the manuscript, are sufficient theoretic bases from which to formulate a testable a priori hypothesis regarding the effect of anodal tDCS over the M1 on motor learning. As the reviewers correctly noted, it is possible that the cathodal influence of the electrode placed on the supra orbital area may have interfered with the test performance. The optimal control experiment would thus have been to increase the size of the cathode to a size where the density of the current would have been small enough to render it functionally inefficient [5].

However, adding such control group (n=20) to the existing data could have introduced a range of confounds. While it would have been possible to draw the sample from the same population as the original experiment, the participants would have been under influences that were not controlled for. For instance, as the participants were university students, influences on the mental state such as time to exams would have been systematically different in the control sample compared to the original sample, making comparison difficult. Furthermore, as the experiment was conducted in an arctic region, where daylight conditions vary between 20 minutes of dim light in December, to 24 hours of sunlight in June, testing the control group at a separate time than the original experiment would have resulted in a control sample that were systematically different in their melatonin cycle as well as level if vitamin D at the time of testing. Finally, changes in the laboratory inventory supply brands on water, conductive paste and rubber electrodes that would have to be custom made in order to be sufficiently large could have made the testing procedure different than the original experiment.

In order to circumnavigate these limitations, a new experiment with 4 groups (n=80) are required, but could not practically be conducted in time for this manuscript. However, I feel that the present experiment still provide valuable empirical evidence on the lack effect of a classical tDCS montage on performance on two common neuropsychological tests.

2. As correctly stated by reviewer 1, the electrode position of the anode was not precisely described in the manuscript. In the experiment, the anode was placed over the C3 or C4 depending on the handedness of the participant. This is now corrected in the manuscript.

3. As pointed out by reviewer 1, TMT performance may be hard to improve with tDCS, as neither Park's dual frontal stimulation, nor our "classic" M1 stimulation did lead to improvements in practice effect. I therefore added a phrase about this to the manuscript.

4. In the introduction we summarize literature on the effect of tDCS on motor outcomes. I am not aware of any studies that demonstrates an effect of tDCS on the specific tests GPT or TMT on healthy subjects with a comparable study design to ours, other than those mentioned. Therefore, the literature from which to draw relevant conclusions is not large. Our results are in line with the consensus article (Ziemann et. al), and I feel a more thorough discussion of the results, for instance by comparing with studies that uses other measures would not add new theoretic insight for the reader.

5. As pointed out by reviewer 1, I would appear likely that body mass did correlate with electric resistance, but in our data BMI did not significant correlate with Impedance (r = .28, p = .23).

6. After thorough consideration, I agree with reviewer 1. This part of the conclusion is not supported by our data, but by the study of Lang et. al (2005), and thus should be removed from the conclusion. I also made corresponding changes in the abstract. However, as our system for registering patient discomfort is designed for to be sensitive for clinical adverse effects, and not strictly discomfort, I retain the discussion about it. This remark is added to the manuscript on page 11, end of 1st paragraph.

Minor

1. As requested, the correction to "new motor skills" has been made.

2. The manuscript now specifies that it refers to behavioral motor and cognitive outcomes. I deleted the confusing and surplus "but more similar in motor domains". However, it is not taken for granted that the effects of anodal are always improved excitability would increase performance. It is merely stated that the facilitation of anodal tDCS is more consistent than inhibition from cathodal in cognitive domains. The rest of the introduction states that the outcome are decided by timing, stimulation parameters, task and even the functional state of the brain (rest vs fatigue) are determinants for the effect of the stimulation. I thus disagree with reviewer that the manuscript contains oversimplifications.

3. The citation is corrected.

4. The requested statement about CNS-acting medication is added to the manuscript. Doses of nicotine is added to table 1. Unfortunately, we did not register data on whether the participants were habitual smokers, or had ceased being so. This is added as a limitation under "limitations".

5. 10 ml of water was the ideal amount to moisten the electrodes without spilling and making the surrounding tissue wet. It is a well tried way of preparing electrodes, see for instance a recent clinical trial [6]. Note that the ability to contain liquid in the swamps will depend on whether they are new or not. For this study, we used only new swamps (supplied by Rogue Solutions), and thus could apply 10ml in every session.

6. As correctly noted by reviewer 1, "after effects" is correct. This is now corrected in the manuscript.

7. As stated by reviewer 1, this is an important distinction between the cited studies, and has consequently been added to the manuscript. I removed the statement about relevance to the present results.

8. All correlations were non-significant. This is now stated in the results.

9. I added the letters linking the analysis in the table to where it occur in the text in a separate column, and bolded the font to improve readability.

Reviewer 2

1. I agree that the order effect might have an effect on the outcomes, and added this statement to the "limitations section". GPT was administered with both tested with both hands as it has normative values for both hands, and are known to be sensitive to lateralization effects from functional brain deficits. GPT assesses eye-hand coordination and motor speed, and is considered a relatively complex task compared to other motor tasks such as Grip Strength and Finger Tapping [7]. Trail making test B is less of a pure motor test, but can be considered a measure of attention, processing speed and mental flexibility that requires complex scanning, and has a motor component [8]. Thus as TMT can be considered less specifically motor focused test than GPT, it was administered with the dominant hand only. As TMT is theoretically thought to tap into a different resource pool than GPT, TMT was added as an alternative area of improvement after tDCS because the time between trials allowed for it.

2. The requested sham vs. control analysis are added to the manuscript. At first, I did not deem them necessary, but since we argue that the real placebo effect is the difference between sham and control, they should be in.

3. I agree with the reviewer that BMI as a predictor for practice effect appear to be novel. I cannot explain this finding, and have stated so in the manuscript. This effect not present on any of the other tests, and might as well be an artifact. However, I feel that the findings regarding caffeine and impedance in the active tDCS group are more interesting for the readers of this article, and would therefore maintain my attempts to explain them. I added some clarifications in this part of the manuscript, that I hope are in line with your request.

Minor

1. I corrected page 2, line 38, and the error on line 62. Moreover, several minor lingual errors were corrected throughout the manuscript.

2. The suggested paper is fits nicely with the part of the introduction that considers inter-individual differences, and is thus added to the manuscript.

3. As requested, nicotine is added to table 1. Number of participants that did not drink coffee, and number of participants that did not use nicotine. A minor comment on the wording: Nicotine is more precise term than smoking since "snus" is commonly used in the population from which the sample is drawn from. Snus is a orally administered wet tobacco that is usually put under the lip. Similar to chewing tobacco.

4. As correctly noted by reviewer 2, "anatomical differences" can be a misleading term, and are changed to "individual differences" or their proper variable names throughout the manuscript.

5. The issue pointed out by reviewer 2 is corrected in the manuscript.

6. The fade in \ fade out time for the sham mode is coded in the study mode of the stimulator to best mimic the sensation of electric stimulation, while minimizing the time the subject is exposed to electric stimulation stimulation. For the active tDCS, the fade in and fade out are set to minimize discomfort. This is a method for sham stimulation that previously have applied in both experimental [9] and clinical [6] studies, and to our experience provide efficient blinding on subjects with no previous experience with tDCS.

7. Sleepiness was registered at the same time as the other adverse effect, following each session. In my view, this is sufficiently described, with reference to Brunoni (2011) under "Materials and Methods" - "Adverse effects".

8. The correlations were conducted as partial correlations with gender as control variable, as gender have a naturally occurring correlation BMI and head circumference, and the outcome tests had gender specific normative scores. No conclusions are drawn from the correlations. The analysis was merely used to detect variables to include in the regression analysis. Thus the threshold for inclusion in the regression were set similarly as the global significance level stated under "statistical analysis" .05. The significance level is now specified in the result section as well.

--

Finally we take the opportunity once again to thank the

reviewers for the constructive criticism of the manuscript. Note that changes to the tables are accompagnied by corresponding changes in the figure captions at the of the manuscript. Revisions in the text are indicted in red fonts.

Yours sincerely,

The authors

1. Nitsche, M.A. and W. Paulus, Excitability changes induced in the human motor cortex by weak transcranial direct current stimulation. The Journal of Physiology, 2000. 527(3): p. 633-639.

2. Nitsche, M.A. and W. Paulus, Sustained excitability elevations induced by transcranial DC motor cortex stimulation in humans. Neurology, 2001. 57(10): p. 1899-901.

3. Nitsche, M.A., et al., Pharmacological Modulation of Cortical Excitability Shifts Induced by Transcranial Direct Current Stimulation in Humans. The Journal of Physiology, 2003. 553(1): p. 293-301.

4. Stagg, C.J. and M.A. Nitsche, Physiological basis of transcranial direct current stimulation. The Neuroscientist, 2011. 17(1): p. 37-53.

5. Nitsche, M.A., et al., Transcranial Direct Current Stimulation: Protocols and Physiological Mechanisms of Action, in Textbook of Neuromodulation2015, Springer. p. 101-111.

6. Fagerlund, A.J., O.A. Hansen, and P.M. Aslaksen, Transcranial direct current stimulation as a treatment for patients with fibromyalgia: a randomized controlled trial. Pain, 2015. 156(1): p. 62-71 10.1016/j.pain.0000000000000006.

7. Merker, B. and K. Podell, Grooved Pegboard Test, in Encyclopedia of Clinical Neuropsychology, J. Kreutzer, J. DeLuca, and B. Caplan, Editors. 2011, Springer New York. p. 1176-1178.

8. Meyers, J., Trail Making Test, in Encyclopedia of Clinical Neuropsychology, J. Kreutzer, J. DeLuca, and B. Caplan, Editors. 2011, Springer New York. p. 2537-2538.

9. Aslaksen, P., O. Vasylenko, and A. Fagerlund, The effect of transcranial direct current stimulation on experimentally induced heat pain. Experimental Brain Research, 2014. 232(6): p. 1865-1873.
